# 3-Hy­droxy-2,2-bis­(1*H*-pyrazol-1-yl)­cyclo­penta­none

**DOI:** 10.1107/S1600536812007659

**Published:** 2012-02-24

**Authors:** Victor B. Rybakov, Anastasia A. Utkina, Alexander V. Kurkin, Marina A. Yurovskaya

**Affiliations:** a Department of Chemistry, Moscow State University, 119992 Moscow, Russian Federation

## Abstract

The title compound, C_11_H_12_N_4_O_2_, was unexpectedly obtained in the reaction of α,α′-disubstituted cyclo­penta­none with 1,1,3,3-tetra­meth­oxy­propane in the presence of dioxane saturated with HCl. It belongs to a previously unknown class of gem-bihetaryl ketones which may be useful for screening of new substances with biological activity. In the studied structure, the cyclo­penta­none moiety adopts an envelope conformation, with the hy­droxy-bearing C atom as the flap [deviation from basal plane = 0.643 (3) Å]. The dihedral angle between the two pyrazole rings is 80.02 (8)°. In the crystal, inversion dimers are formed *via* a pair of O—H⋯N hydrogen bonds.

## Related literature

For the medicinal chemistry of chiral carbo- and heterocyclic substituents of pyrazole, see: Bennani *et al.* (2007 ▶); Srivastava *et al.* (2007 ▶). For the α-amination of carbonyl compounds, see: List (2002 ▶). For standard values of bond lengths in organic compounds, see: Allen *et al.* (1987 ▶).
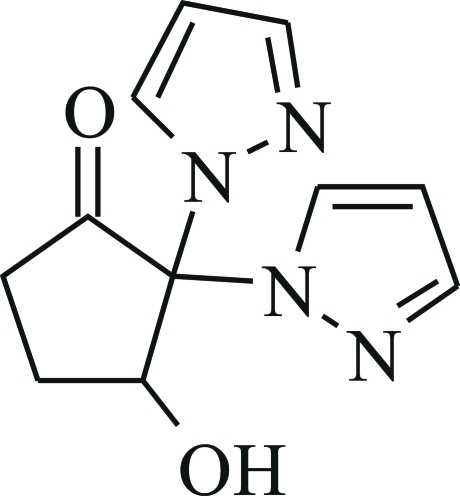


## Experimental

### 

#### Crystal data

C_11_H_12_N_4_O_2_
*M* *_r_* = 232.25
Monoclinic, 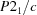

*a* = 11.4360 (11) Å
*b* = 9.5925 (9) Å
*c* = 11.5968 (11) Å
β = 117.25 (2)°
*V* = 1131.0 (3) Å^3^
*Z* = 4
Ag *K*α radiation
λ = 0.56085 Å
μ = 0.06 mm^−1^
*T* = 295 K
0.20 × 0.20 × 0.20 mm

#### Data collection

Enraf–Nonius CAD-4 diffractometer
2709 measured reflections
2458 independent reflections
1723 reflections with *I* > 2σ(*I*)
*R*_int_ = 0.026
1 standard reflections every 60 min intensity decay: none

#### Refinement

*R*[*F* ^2^ > 2σ(*F* ^2^)] = 0.046
*wR*(*F* ^2^) = 0.124
*S* = 1.04
2458 reflections
158 parameters
H atoms treated by a mixture of independent and constrained refinement
Δρ_max_ = 0.18 e Å^−3^
Δρ_min_ = −0.22 e Å^−3^

### 

Data collection: *CAD-4 EXPRESS* (Enraf–Nonius, 1994 ▶); cell refinement: *CAD-4 EXPRESS*; data reduction: *XCAD4* (Harms & Wocadlo, 1995 ▶); program(s) used to solve structure: *SHELXS97* (Sheldrick, 2008 ▶); program(s) used to refine structure: *SHELXL97* (Sheldrick, 2008 ▶); molecular graphics: *ORTEP-3* (Farrugia, 1997 ▶); software used to prepare material for publication: *WinGX* (Farrugia, 1999 ▶).

## Supplementary Material

Crystal structure: contains datablock(s) global, I. DOI: 10.1107/S1600536812007659/yk2045sup1.cif

Structure factors: contains datablock(s) I. DOI: 10.1107/S1600536812007659/yk2045Isup2.hkl

Supplementary material file. DOI: 10.1107/S1600536812007659/yk2045Isup3.cml

Additional supplementary materials:  crystallographic information; 3D view; checkCIF report

## Figures and Tables

**Table 1:** Hydrogen-bond geometry (Å, °)

*D*—H⋯*A*	*D*—H	H⋯*A*	*D*⋯*A*	*D*—H⋯*A*
O3—H3*a*⋯N22*b*^i^	0.90 (3)	1.88 (3)	2.781 (2)	179 (2)

Symmetry code: (i) 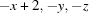.

**Fig. 1.**
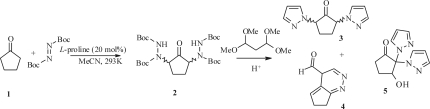
Synthetic path for title compound.

**Fig. 2.**
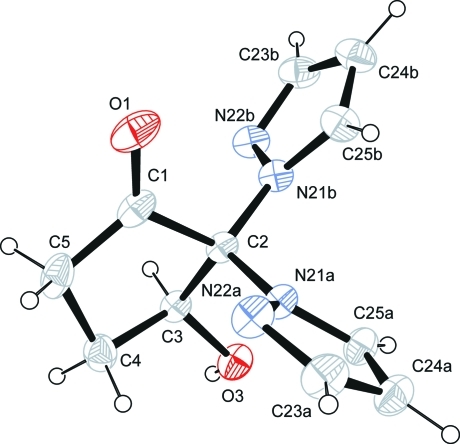
The structure of the title molecule with the atom numbering scheme. Displacement ellipoids are drawn at the 30% probability level. H atoms are presented as small spheres of arbitrary radius.

## supplementary crystallographic information

### Comment

The pyrazole derivatives with chiral carbo- and heterocyclic substituents at the nitrogen atom have great importance for medicinal chemistry (Bennani *et al.*, 2007; Srivastava *et al.*, 2007). The substituted hydrazine derivatives are suitable and accessible reagents in the reactions with 1,3-dicarbonilyl compounds or their masked forms for the preparation of various N-substituted pyrazoles. We have used for the synthesis of starting hydrazine the reaction of direct stereoselective α-amination of cyclopentanone catalyzed by *L*-proline with azadicarboxylates as the source of nitrogen (List, 2002). Under these conditions the reaction of α-amination affords to bis-α,α'-aminated ketone derivative 2 (Fig. 1) as a main product, which was transformed to 2,5-di-1*H*-pyrazol-1-ylcyclopentanone 3 (Fig. 1) by further cyclization with 1,1,3,3-tetramethoxypropane. However, in reaction mixture we have found also two unexpected compounds 4 and 5 (Fig. 1). Formation of the compound 4 can be explained by the competitive intramolecular cyclization of 2 with the participation of ketone group. Appearance of compound 5, which structure was determined by *X*-ray analysis, is totally unexpected and unusual. It is assumed that such product results from the unusual intermediate formed *via* uncommon α,α-diamination, that hasn't been previously described, instead of usual α,α'-diamination. The mechanism of formation of 5 is currently under investigation and will be discussed in a further paper.

Compound 5 was obtained by chromatographic separation of complex reaction mixture formed due to the catalyzed by *L*-proline α-amination of cyclopentanone 1 (Fig. 1) with azadicarboxylates. Chromatographic separation was carried out using a combination of column with silica gel and *PTLC*. A gradient elution system was developed enabling the resolution of mixture of compounds 4 and 5 and pure product 2,5-di-(1*H*-pyrazol-1-yl)cyclopentanone 3. Further *PTLC* of mixture of compounds 4 and 5 afforded to obtain both pure products as individual compounds.

In the title compound (Fig. 2), two essentially planar pyrazole rings (largest deviations from l.s. planes 0.002 (2) and 0.007 (1) Å) form dihedral angle of 80.02 (8)°. Five-membered cyclopentanone ring has envelope conformation with the C3 atom as a flap (deviation from the plane C1/C2/C4/C5 0.643 (3) Å). All bond lengths are within expected ranges (Allen *et al.*, 1987).

In the crystal, title molecules form centrosymmetric dimers by intermolecular H-bonds O3–H3a···N22*b*^i^ with parameters: O3–H3a = 0.90 (3) Å, H3a···N22*b*^i^ = 1.88 (3) Å, O3···N22*b*^i^ = 2.781 (2) Å and angle O3–H3a···N22*b*^i^ = 179 (2)°. Symmetry code: (i) -*x* + 2, -*y*, -*z*.

### Experimental

Tetra-*tert*-butyl 1,1'-(2-oxocyclopentane-1,3-diyl)dihydrazine-1,2-dicarboxylate 2 was prepared by following procedure: a solution of di-*tert*-butyl (*E*)-diazene-1,2-dicarboxylate (1 g, 4.3 mmol) and *L*-proline (0.5 g, 0.43 mmol) in CH_3_CN (43 ml) was cooled to 273 K and cyclopentanone (0.64 ml, 6.5 mmol) was added dropwise. The reaction mixture was stirred at 273 K for 24 h, and allowed to warm slowly to room temperature. After 1 h, the mixture was concentrated and the crude residue was purified by column chromatography on silica gel (eluent - petroleum ether: ethyl acetate 5: 1) to afford 1.92 g (61% yield) of required product as a white foam. Spectrum ^1^H NMR (400 MHz, CDCl_3_), δ: 1.44 (36*H*, s, 4 C(CH_3_)_3_); 1.74-2.07 (2*H*, m, CH_2_); 2.13-2.52 (2*H*, m, CH_2_); 4.10 and 4.42 (both 1H, 2 br. s, CH); 6.14 and 6.44 (both 1H, 2 br. s, NH). Spectrum ^13^C NMR, (400 MHz, CDCl_3_) δ: 28.0; 28.1; 40.2; 45.0; 54.6; 57.7; 58.2; 80.7; 81.6; 154.8; 155.3; 205.1. MS (ESI), *m/z* (%): 545 [*M*+H]^+^ (0.1), 450 (5), 277 (27), 157 (100), 138 (14). MS (EI, 70 eV), *m/z* (%): 276 (46), 157 (47), 102 (45), 57 (100). Anal. Calculated for C_25_H_44_N_4_O_9_: C 55.13, H 8.14, N 10.29. Found: C 55.28, H 8.20, N 10.07.

General procedure for synthesis of 3, 4 and 5. The compound 2 (0.76 g, 1.2 mmol) was dissolved in dioxane (5 ml), and a saturated solution of HCl in dioxane (~12%, 1.82 g, 5 eq.) was added and stirred for 0.5 h. Than 1,1,3,3-tetramethoxypropane (0.59 g, 3.6 mmol, 3 eq.) was added, and the reaction mixture was left at room temperature overnight. Further it was concentrated to dryness under reduced pressure, the residue dissolved in CH_2_Cl_2_ (20 ml) and quenched with saturated NaHCO_3_. The aqueous layers were back-extracted with CH_2_Cl_2_ (3×15 ml). The combined organic layers were dried over Na_2_SO_4_, filtered, and concentrated to dryness under reduced pressure. The residue was purified by column chromatography on silica gel (eluent - petroleum ether: ethyl acetate 3:1) to afford 0.11 g (15% yield) of required 2,5-di-(pyrazol-1*H*-yl)cyclopentanone 3 as a light yellow oil. Spectrum ^1^H NMR (400 MHz, CDCl_3_), δ: 2.22-2.32 (2*H*, m, CH_2_); 2.34-2.45 (2*H*, m, CH_2_); 5.31 (2*H*, m, CH_2_); 6.36-6.42 (2*H*, m, H-4 pyrazole); 7.65-7.73 (2*H*, m, H-5 pyrazole); 7.86-9.92 (2*H*, m, H-3 pyrazole). Spectrum ^13^C NMR, (400 MHz, CDCl_3_), δ: 29.7(2 C); 61.2(2 C); 109.2; 125.6; 133.5; 208.1. MS (ESI), *m/z* (%): 217 [*M*+H]^+^ (1), 149 (100). Anal. Calculated for C_11_H_12_N_4_O: C 61.10; H 5.59, N 25.91. Found: C 59.98; H 5.47; N 25.82.

Further *PTLC* of mixture of compounds 4 and 5, using a 10:1 mixture of petroleum ether and methanole as eluent, gave both pure products as individual compounds with yelds 18% and 13%, respectively.

The 6,7-dihydro-4*H*-cyclopenta[*c*]pyridazine-4-carbaldehyde 4: a colourless oil. Spectrum ^1^H NMR (400 MHz, CDCl_3_), δ: 2.65-2.70 (2*H*, m, CH_2_), 2.73-2.79 (2*H*, m, CH_2_), 6.38 (1*H*, dd, *J*_1_ = 1.8, *J*_2_ = 2.5, H-4), 7.67 (1*H*, d, *J*_1_ = 1.5 H-3), 7.87 (1*H*, t, *J*_1_ = 3.1 H-5), 8.54 (1*H*, dd, *J*_1_ = 0.36, *J*_2_ = *J*_3_ = 2.56, CHO). Spectrum ^13^C NMR, (400 MHz, CDCl_3_), δ: 23.93, 35.18, 106.75, 128.68, 141.26, 146.00, 148.15, 200.75. HRMS (ESI, 4,5 mV). Calculated for C_8_H_8_N_2_O: 148.0631, Found, *m/z*: 148.0636 [*M*+H]^+^.

The 3-hydroxy-2,2-di-(pyrazol-1*H*-yl)cyclopentanone 5: light yellow solid. *M*.p. 385-386 K (decomp.). Spectrum ^1^H NMR (400 MHz, CDCl_3_), δ: 1.98-2.08 (1*H*, m, CH_2_), 2.09-2.18 (1*H*, m, CH_2_), 2.70 and 2.65 (0.60 H and 0.40 H, both ddd, *J*_1_ = 9.3, *J*_2_ = 4.6, *J*_3_ = 1/2, CH_2_), 2.89 and 2.84 (0.35 H and 0.65 H, both ddd, *J*_1_ = 9.3, *J*_2_ = 7.7, *J*_3_ = 0.6, CH_2_), 4.88 (1*H*, br. s, OH), 5.25 (1*H*, t, *J* = 4.6 CHOH), 6.34-6.37 (2*H*, m, H-4,4' pyrazole), 7.49 (1*H*, dd, *J*_1_ = 2.6, *J*_2_ = 0.6, H-5 pyrazole), 7.58 (1*H*, dd, *J*_1_ = 1.8, *J*_2_ = 1/2, H-5' pyrazole), 7.62 (1*H*, dd, *J*_1_ = 1.8, *J*_2_ = 1/2, H-3 pyrazole), 7.67 (1*H*, dd, *J*_1_ = 2.6, *J*_2_ = 0.6, H-3' pyrazole). Spectrum ^13^C NMR, (400 MHz, CDCl_3_), δ: 24.96, 34.11, 76.03, 94.68, 107.07, 107.44, 128.44, 130.69, 140.18, 140.21, 203.57. MS (EI, 70 eV), *m/z* (%): 165 [*M*^+^ - *Pyr*] (62), 137 (22), 119 (72), 95 (100), 81 (22), 69 (18). Anal. Calculated for C_11_H_12_N_4_O_2_: C 56.89; H 5.21, N 24.12. Found: C 56.40; H 5.68; N 23.98.

The single crystals of title compound suitable for *X*-ray analysis were grown from methanol solution by slow evaporation at room temperature.

### Refinement

C-bound H atoms were placed in calculated positions with C–H 0.93-0.98 Å and refined as riding with *U*_iso_(H) = 1.2(1.5)*U*_eq_(C). The O-bound H atom forming hydrogen bond was located from difference Fourier map and refined independently.

### Crystal data

**Table:**

C_11_H_12_N_4_O_2_	*F*(000) = 488
*M**_r_* = 232.25	*D*_x_ = 1.364 Mg m^−^^3^
Monoclinic, *P*2_1_/*c*	Melting point = 385–386 K
Hall symbol: -P 2ybc	Ag *K*α radiation, λ = 0.56085 Å
*a* = 11.4360 (11) Å	Cell parameters from 25 reflections
*b* = 9.5925 (9) Å	θ = 10.0–12.0°
*c* = 11.5968 (11) Å	µ = 0.06 mm^−^^1^
β = 117.25 (2)°	*T* = 295 K
*V* = 1131.0 (3) Å^3^	Prism, light yellow
*Z* = 4	0.20 × 0.20 × 0.20 mm

### Data collection

**Table:**

Enraf–Nonius CAD-4 diffractometer	*R*_int_ = 0.026
Radiation source: fine-focus sealed tube	θ_max_ = 21.0°, θ_min_ = 1.6°
Graphite monochromator	*h* = −14→12
non–profiled ω scans	*k* = 0→12
2709 measured reflections	*l* = 0→14
2458 independent reflections	1 standard reflections every 60 min
1723 reflections with *I* > 2σ(*I*)	intensity decay: none

### Refinement

**Table:**

Refinement on *F*^2^	Primary atom site location: structure-invariant direct methods
Least-squares matrix: full	Secondary atom site location: difference Fourier map
*R*[*F*^2^ > 2σ(*F*^2^)] = 0.046	Hydrogen site location: inferred from neighbouring sites
*wR*(*F*^2^) = 0.124	H atoms treated by a mixture of independent and constrained refinement
*S* = 1.04	*w* = 1/[σ^2^(*F*_o_^2^) + (0.0634*P*)^2^ + 0.119*P*] where *P* = (*F*_o_^2^ + 2*F*_c_^2^)/3
2458 reflections	(Δ/σ)_max_ < 0.001
158 parameters	Δρ_max_ = 0.18 e Å^−^^3^
0 restraints	Δρ_min_ = −0.22 e Å^−^^3^

### Special details

**Table:**

Geometry. All s.u.'s (except the s.u. in the dihedral angle between two l.s. planes) are estimated using the full covariance matrix. The cell s.u.'s are taken into account individually in the estimation of s.u.'s in distances, angles and torsion angles; correlations between s.u.'s in cell parameters are only used when they are defined by crystal symmetry. An approximate (isotropic) treatment of cell s.u.'s is used for estimating s.u.'s involving l.s. planes.
Refinement. Refinement of *F*^2^ against ALL reflections. The weighted *R*-factor w*R* and goodness of fit *S* are based on *F*^2^, conventional *R*-factors *R* are based on *F*, with *F* set to zero for negative *F*^2^. The threshold expression of *F*^2^ > 2σ(*F*^2^) is used only for calculating *R*-factors(gt) *etc*. and is not relevant to the choice of reflections for refinement. *R*-factors based on *F*^2^ are statistically about twice as large as those based on *F*, and *R*-factors based on ALL data will be even larger.

### Fractional atomic coordinates and isotropic or equivalent isotropic displacement parameters (Å^2^)

**Table:**

	*x*	*y*	*z*	*U*_iso_*/*U*_eq_	
C1	0.8285 (2)	0.02907 (18)	0.22875 (18)	0.0442 (5)	
O1	0.74464 (17)	−0.04122 (16)	0.23414 (15)	0.0664 (5)	
C2	0.81692 (16)	0.09021 (17)	0.09920 (15)	0.0334 (4)	
C3	0.96176 (17)	0.10037 (18)	0.12975 (15)	0.0366 (4)	
H3	0.9944	0.0069	0.1259	0.044*	
O3	0.97820 (14)	0.18725 (14)	0.04063 (13)	0.0470 (4)	
H3a	1.054 (3)	0.162 (2)	0.040 (2)	0.074 (7)*	
C4	1.02716 (19)	0.1504 (2)	0.26969 (17)	0.0479 (5)	
H4a	1.0111	0.2490	0.2748	0.058*	
H4b	1.1213	0.1343	0.3099	0.058*	
C5	0.9623 (2)	0.0629 (2)	0.33416 (17)	0.0540 (5)	
H5a	1.0122	−0.0216	0.3706	0.065*	
H5b	0.9563	0.1150	0.4029	0.065*	
N21a	0.75682 (14)	0.22639 (14)	0.08142 (13)	0.0355 (3)	
N22a	0.72893 (16)	0.27771 (16)	0.17473 (15)	0.0467 (4)	
C23a	0.6800 (2)	0.4024 (2)	0.1306 (2)	0.0574 (6)	
H23a	0.6514	0.4639	0.1744	0.069*	
C24a	0.6759 (2)	0.4315 (2)	0.0121 (2)	0.0547 (5)	
H24a	0.6452	0.5123	−0.0369	0.066*	
C25a	0.72630 (18)	0.31694 (19)	−0.01749 (18)	0.0442 (4)	
H25a	0.7376	0.3034	−0.0912	0.053*	
N21b	0.73500 (14)	0.00404 (14)	−0.01115 (13)	0.0368 (3)	
N22b	0.78713 (15)	−0.10826 (15)	−0.04212 (15)	0.0428 (4)	
C23b	0.6833 (2)	−0.1781 (2)	−0.1270 (2)	0.0532 (5)	
H23b	0.6885	−0.2604	−0.1668	0.064*	
C24b	0.5666 (2)	−0.1145 (2)	−0.1493 (2)	0.0550 (5)	
H24b	0.4817	−0.1443	−0.2044	0.066*	
C25b	0.60277 (18)	0.0009 (2)	−0.07340 (18)	0.0481 (5)	
H25b	0.5464	0.0660	−0.0658	0.058*	

### Atomic displacement parameters (Å^2^)

**Table:**

	*U*^11^	*U*^22^	*U*^33^	*U*^12^	*U*^13^	*U*^23^
C1	0.0694 (12)	0.0345 (9)	0.0456 (10)	0.0067 (9)	0.0409 (10)	0.0039 (8)
O1	0.0940 (12)	0.0587 (9)	0.0741 (10)	−0.0051 (8)	0.0623 (10)	0.0126 (8)
C2	0.0427 (9)	0.0331 (8)	0.0326 (8)	0.0013 (7)	0.0242 (7)	−0.0004 (7)
C3	0.0436 (9)	0.0355 (9)	0.0368 (9)	0.0037 (7)	0.0238 (8)	0.0013 (7)
O3	0.0464 (7)	0.0543 (8)	0.0542 (8)	0.0036 (6)	0.0350 (7)	0.0106 (6)
C4	0.0529 (12)	0.0481 (11)	0.0393 (10)	0.0043 (9)	0.0180 (9)	−0.0022 (8)
C5	0.0798 (15)	0.0514 (12)	0.0347 (10)	0.0157 (11)	0.0297 (10)	0.0068 (9)
N21a	0.0444 (8)	0.0353 (8)	0.0371 (7)	0.0049 (6)	0.0275 (6)	0.0014 (6)
N22a	0.0625 (10)	0.0443 (9)	0.0483 (9)	0.0085 (7)	0.0385 (8)	−0.0037 (7)
C23a	0.0696 (14)	0.0443 (11)	0.0681 (14)	0.0122 (10)	0.0400 (12)	−0.0082 (10)
C24a	0.0587 (13)	0.0420 (11)	0.0646 (13)	0.0100 (9)	0.0292 (11)	0.0103 (10)
C25a	0.0496 (10)	0.0450 (10)	0.0440 (10)	0.0053 (8)	0.0266 (9)	0.0084 (8)
N21b	0.0420 (8)	0.0385 (8)	0.0399 (8)	0.0002 (6)	0.0273 (7)	−0.0042 (6)
N22b	0.0503 (9)	0.0394 (8)	0.0510 (9)	0.0012 (7)	0.0338 (8)	−0.0067 (7)
C23b	0.0663 (13)	0.0465 (11)	0.0574 (12)	−0.0122 (10)	0.0373 (11)	−0.0137 (9)
C24b	0.0500 (11)	0.0651 (13)	0.0543 (11)	−0.0165 (10)	0.0279 (10)	−0.0114 (10)
C25b	0.0417 (10)	0.0581 (12)	0.0534 (11)	−0.0016 (9)	0.0293 (9)	−0.0052 (10)

### Geometric parameters (Å, º)

**Table:**

N21a—C25a	1.351 (2)	C4—C5	1.523 (3)
N21a—N22a	1.3531 (19)	C4—H4a	0.9700
N21a—C2	1.446 (2)	C4—H4b	0.9700
N22a—C23a	1.321 (2)	C5—C1	1.493 (3)
C23a—C24a	1.382 (3)	C5—H5a	0.9700
C23a—H23a	0.9300	C5—H5b	0.9700
C24a—C25a	1.356 (3)	C1—O1	1.197 (2)
C24a—H24a	0.9300	N21b—C25b	1.345 (2)
C25a—H25a	0.9300	N21b—N22b	1.3568 (18)
C2—N21b	1.449 (2)	N22b—C23b	1.325 (2)
C2—C3	1.530 (2)	C23b—C24b	1.380 (3)
C2—C1	1.561 (2)	C23b—H23b	0.9300
C3—O3	1.406 (2)	C24b—C25b	1.356 (3)
C3—C4	1.521 (2)	C24b—H24b	0.9300
C3—H3	0.9800	C25b—H25b	0.9300
O3—H3a	0.90 (3)		
			
C25a—N21a—N22a	112.41 (14)	C3—C4—H4a	111.0
C25a—N21a—C2	128.53 (14)	C5—C4—H4a	111.0
N22a—N21a—C2	119.03 (13)	C3—C4—H4b	111.0
C23a—N22a—N21a	103.46 (15)	C5—C4—H4b	111.0
N22a—C23a—C24a	112.56 (17)	H4a—C4—H4b	109.0
N22a—C23a—H23a	123.7	C1—C5—C4	105.35 (14)
C24a—C23a—H23a	123.7	C1—C5—H5a	110.7
C25a—C24a—C23a	105.26 (17)	C4—C5—H5a	110.7
C25a—C24a—H24a	127.4	C1—C5—H5b	110.7
C23a—C24a—H24a	127.4	C4—C5—H5b	110.7
N21a—C25a—C24a	106.31 (16)	H5a—C5—H5b	108.8
N21a—C25a—H25a	126.8	O1—C1—C5	128.69 (18)
C24a—C25a—H25a	126.8	O1—C1—C2	123.02 (18)
N21a—C2—N21b	108.45 (13)	C5—C1—C2	108.04 (15)
N21a—C2—C3	111.65 (13)	C25b—N21b—N22b	111.25 (14)
N21b—C2—C3	115.74 (13)	C25b—N21b—C2	126.87 (14)
N21a—C2—C1	107.57 (12)	N22b—N21b—C2	120.12 (14)
N21b—C2—C1	111.79 (14)	C23b—N22b—N21b	104.18 (15)
C3—C2—C1	101.26 (13)	N22b—C23b—C24b	112.13 (18)
O3—C3—C4	115.56 (15)	N22b—C23b—H23b	123.9
O3—C3—C2	111.26 (14)	C24b—C23b—H23b	123.9
C4—C3—C2	102.63 (13)	C25b—C24b—C23b	104.93 (18)
O3—C3—H3	109.0	C25b—C24b—H24b	127.5
C4—C3—H3	109.0	C23b—C24b—H24b	127.5
C2—C3—H3	109.0	N21b—C25b—C24b	107.48 (17)
C3—O3—H3a	107.6 (15)	N21b—C25b—H25b	126.3
C3—C4—C5	103.75 (15)	C24b—C25b—H25b	126.3
			
C25a—N21a—N22a—C23a	0.1 (2)	C4—C5—C1—O1	177.50 (19)
C2—N21a—N22a—C23a	178.13 (16)	C4—C5—C1—C2	3.11 (19)
N21a—N22a—C23a—C24a	0.1 (2)	N21a—C2—C1—O1	90.9 (2)
N22a—C23a—C24a—C25a	−0.3 (3)	N21b—C2—C1—O1	−28.1 (2)
N22a—N21a—C25a—C24a	−0.3 (2)	C3—C2—C1—O1	−151.91 (18)
C2—N21a—C25a—C24a	−178.07 (16)	N21a—C2—C1—C5	−94.37 (16)
C23a—C24a—C25a—N21a	0.4 (2)	N21b—C2—C1—C5	146.68 (15)
C25a—N21a—C2—N21b	−58.2 (2)	C3—C2—C1—C5	22.86 (17)
N22a—N21a—C2—N21b	124.20 (15)	N21a—C2—N21b—C25b	−38.9 (2)
C25a—N21a—C2—C3	70.5 (2)	C3—C2—N21b—C25b	−165.28 (16)
N22a—N21a—C2—C3	−107.11 (16)	C1—C2—N21b—C25b	79.5 (2)
C25a—N21a—C2—C1	−179.25 (17)	N21a—C2—N21b—N22b	157.55 (13)
N22a—N21a—C2—C1	3.1 (2)	C3—C2—N21b—N22b	31.2 (2)
N21a—C2—C3—O3	−49.79 (17)	C1—C2—N21b—N22b	−84.02 (17)
N21b—C2—C3—O3	74.91 (18)	C25b—N21b—N22b—C23b	1.36 (19)
C1—C2—C3—O3	−164.01 (13)	C2—N21b—N22b—C23b	167.26 (15)
N21a—C2—C3—C4	74.37 (16)	N21b—N22b—C23b—C24b	−0.9 (2)
N21b—C2—C3—C4	−160.92 (14)	N22b—C23b—C24b—C25b	0.2 (2)
C1—C2—C3—C4	−39.84 (16)	N22b—N21b—C25b—C24b	−1.3 (2)
O3—C3—C4—C5	164.39 (15)	C2—N21b—C25b—C24b	−166.00 (16)
C2—C3—C4—C5	43.13 (18)	C23b—C24b—C25b—N21b	0.6 (2)
C3—C4—C5—C1	−28.36 (19)		

### Hydrogen-bond geometry (Å, º)

**Table:**

*D*—H···*A*	*D*—H	H···*A*	*D*···*A*	*D*—H···*A*
O3—H3*a*···N22*b*^i^	0.90 (3)	1.88 (3)	2.781 (2)	179 (2)

Symmetry code: (i) −*x*+2, −*y*, −*z*.
